# A critical role of outer membrane vesicles in antibiotic resistance in carbapenem-resistant *Klebsiella pneumoniae*

**DOI:** 10.1186/s12941-023-00645-4

**Published:** 2023-11-02

**Authors:** Lifeng Yao, Beiwen Wei, Yuanxia Wang, Beihui Xu, Meng Yang, Xu Chen, Fuxiang Chen

**Affiliations:** 1 Department of Laboratory Medicine, Shanghai Ninth People’s Hospital, Shanghai Jiao Tong University School of Medicine, Shanghai, China; 2 Faculty of Medical Laboratory Science, College of Health Science and Technology, Shanghai Jiao Tong University School of Medicine, Shanghai, China

**Keywords:** Carbapenem-resistant *Klebsiella pneumoniae*, Outer membrane vesicles, Carbapenem-resistant *Enterobacterales*, Antibiotic resistance

## Abstract

**Background:**

This study aimed to illustrate the status of carbapenem-resistant *Enterobacterales* (CRE) infections in a Chinese tertiary hospital and to investigate the role of outer membrane vesicles (OMVs) in antibiotic resistance in carbapenem-resistant *Klebsiella pneumoniae* (CRKP).

**Methods:**

The data of CRE infections was collected from laboratory records, and the CRE isolates from two distinct periods (2015/07 to 2017/07 and 2020/04 to 2021/04) were enrolled to detect the carbapenemase genes by polymerase chain reaction (PCR). Multilocus sequence typing (MLST) was used to analyze the molecular characterization of CRKP. The conjugation assay was performed to verify the transmission of the antibiotic resistance plasmid. The OMVs of CRKP were isolated with a method combining an electrophoretic technique with a 300 kDa cut-off dialysis bag. The protein components in CRKP OMVs were analyzed by liquid chromatography tandem-mass spectrometry (LC–MS/MS), and the meropenem-hydrolyzing bioactivity of KPC in CRKP OMVs was determined with different treatments in vitro.

**Results:**

A total of 178 CRE isolates, including 100 isolates from 2015/07 to 2017/07 and 78 isolates from 2020/04 to 2021/04, were collected for the detection of carbapenemase genes. We found that the carbapenemase gene blaKPC was the most prevalent, followed by blaNDM. By MLST, we found that sequence type (ST) 11 CRKP (96.1%) was the leading type during 2015/07 to 2017/07 and that the ST15 CRKP increased to 46.2% in the late period of 2020/04 to 2021/04. The diameters of *Klebsiella pneumoniae* OMVs ranged from 100 to 200 nm, and by proteomics analysis the most proteins from OMVs belonged to the “enzyme” group. The KPC enzyme was found in the OMVs from CRKP, and the OMVs could protect inside KPC from proteinase K digestion. Moreover, the KPC enzymes within OMVs, which could be released after Triton X-100 treatment, could hydrolyze meropenem.

**Conclusions:**

CRE has increasingly caused infections in hospitals, and blaKPC-positive CRKP infections have constituted a major proportion of infections in the past decade. The OMVs play a critical role in antibiotic resistance in CRKP.

## Highlights

- Prevalence of ST11 blaKPC-positive CRKP in a tertiary hospital from Shanghai
- Co-existence of ST11 and ST15 CRKP
- Isolation of OMVs from CRKP culture supernatant by ELD
- A critical role of OMVs in antibiotic resistance in CRKP

## Introduction

The global prevalence of multidrug-resistant organisms (MDROs) in nosocomial settings has posed a great burden on public health, and the continuing increase of carbapenem-resistant *Enterobacterales* (CRE) is associated with significant morbidity and mortality [1]. CRE infections are difficult to treat as they are resistant to commonly used (or even all available) antibiotics. The production of carbapenemases is the main antibiotic resistance mechanism developed by CRE, and the common carbapenemases found in CRE strains include *Klebsiella pneumoniae* carbapenemase (KPC), verona vntegron-encoded metallo-beta-lactamase (VIM), imipenemase (IMP), New Delhi metallo-beta-lactamase (NDM), oxacillinase-48 (OXA-48) and OXA-181 [1, 2]. Different carbapenemase-producing CRE strains are predominant in distinct countries, and blaKPC and blaNDM are the two major carbapenemase genes in CRE strains from China [3]. Identification of the carbapenemase type would contribute to the treatment of CRE infections. Examples of bacteria among CRE include *Klebsiella pneumoniae* (*K*. *pneumoniae*) and *Escherichia coli* (*E*. *coli*).

**Fig. 1:**
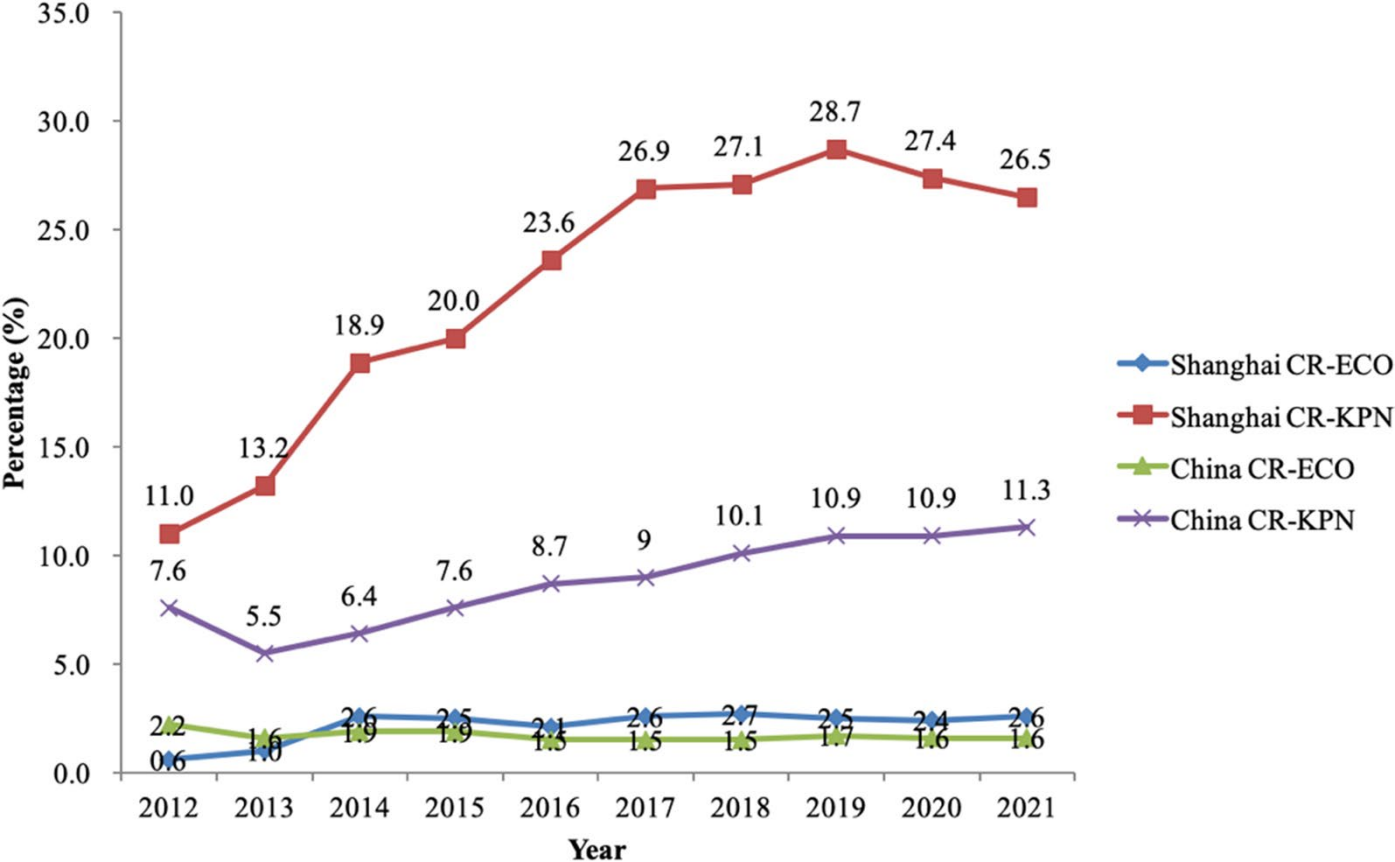
The trend of carbapenem-resistant *Klebsiella pneumoniae* (CR-KPN) and carbapenem-resistant *Escherichia coli* (CR-ECO) in China and in Shanghai

In China, carbapenem-resistant *K*. *pneumoniae* (CRKP) has been the most prevalent species, followed by *E*. *coli* [3, 4]. According to the data from China Antimicrobial Resistance Surveillance System, the isolation rate of CRKP in China increased from 7.6% in 2012 to 11.3% in 2021. Similarly, the isolation rate of CRKP in Shanghai has increased to 26.5% in 2021, more than two times that in 2012. In the meanwhile, the isolation rate of carbapenem-resistant *E*. *coli* in Shanghai increased from 0.6% in 2012 to 2.6% in the year of 2021 despite the stable isolation of carbapenem-resistant *E*. *coli* in China (Fig. 1). A systematic study revealed that the majority of CRKP in China belonged to the sequence type (ST) 11 [3]. Moreover, the emergence of hypervirulent CRKP belonging to ST11-K47/K64, which could cause liver abscesses, further complicated clinical practice [5, 6]. Therefore, much more work should be done to provide insights into the control and treatment of CRE infections.

Extracellular vesicles (EVs) are membrane-derived lipid bilayers secreted by living cells including bacteria [7]. In Gram-negative bacteria, EVs are pinched off from the outer membrane and are called outer membrane vesicles (OMVs) [8]. OMVs, secreted during the growth of all Gram-negative bacteria, are spherical vesicles with a particle size of 20–400 nm [9]. OMVs have recently attracted increasing attention in the field of microbial research. Through carrying bacteria-derived proteins, lipids, nucleic acids and other substances, OMVs are widely involved in biological information transmission, killing competitive bacteria and exerting drug resistance [10, 11]. Ciofu et al. first reported the existence of related extended-spectrum beta-lactamase (ESBL) in OMVs [12], and antibiotic resistant strains can shield susceptible strains from antibiotics by secreting OMVs, which carry antibiotic-inactivating enzymes [13]. Recently, Zhang et al. proved that carbapenemase-loaded OMVs could protect *Pseudomonas aeruginosa* by degrading imipenem and promoting mutation of the antimicrobial resistance gene OprD [14]. In the present study, we analyzed the prevalence of CRE in a Chinese tertiary hospital and the carbapenemases in CRE isolates from two different periods. Moreover, the molecular types of CRKP were assessed. Finally, the proteomic profiles of OMVs isolated from CRKP were investigated, and the role of OMVs in antibiotic resistance was also explored.

## Materials and methods

### Data collection

Data on the carbapenem-resistant *K*. *pneumoniae* and carbapenem-resistant *E*. *coli* in China and Shanghai were obtained from China Antimicrobial Resistance Surveillance System (CARSS, http://www.carss.cn/), and the data of CRE infections in a tertiary hospital (Shanghai Ninth People’s Hospital) were collected from laboratory records. This tertiary hospital, which is known for stomatology, plastic surgery and treatment of inflammatory bowel disease, locates in Shanghai with 2150 beds and 1000 dental chairs, serving more than 3 million outpatients and 90,000 admissions per year.

### Bacterial isolates

The CRE isolates were identified by VITEK 2 COMPACT (BioMérieux, France) or MALDI-TOF MS (BioMérieux, France). The antimicrobial susceptibility testing of the isolates to clinically common antibiotics was performed using a GN13 Gram-negative bacterial antimicrobial susceptibility card (BioMérieux, France) and the disk diffusion method. The CRE isolates were preserved in nutrient broth containing 30% glycerol. The isolates were included when CRE was isolated from (1) a normally sterile site such as blood, central venous catheter, bile, urine and so on, or (2) from a nonsterile site with an infection history such as sputum and wound abscess. The isolates would be excluded when (1) duplicate isolate was isolated from the same patient, (2) the isolate was from screening samples such as nasal swab and anal swab, or (3) the clinical information was missing.

### Detection of carbapenemase genes

The CRE isolates from two distinct periods (2015/07 to 2017/07 and 2020/04 to 2021/04) were recovered, and the DNA was extracted. Detection of five common carbapenemase genes, including blaKPC, blaNDM, blaIMP, blaVIM and blaOXA was performed by PCR-based methods as previously described [15].

### Sequence typing of CRKP, plasmid conjugation assay and whole genome sequencing

Multilocus sequence typing (MLST) of CRKP was performed. Briefly, the internal fragments of *K*. *pneumoniae* housekeeping genes, including rpoB, gapA, mdh, pgi, infB, phoE and tonB, were amplified by PCR [16], and the amplification product was sequenced by Shanghai Sangon Biotech. The sequence type (ST) was obtained at https://bigsdb.pasteur.fr/cgi-bin/bigsdb/bigsdb.pl?db=pubmlst_klebsiella_seqdef&page=profiles after allelic profiling of housekeeping genes.

The plasmid conjugation assay of CRKP was carried out with the *E*. *coli* EC600 used as the recipient strain. Briefly, the donor and the recipient strains were co-cultured, and then the conjugated EC600 was screened by the Luria–Bertani (LB) agar plate containing meropenem (0.8 μg/mL, Selleck, UK) and rifampicin (300 μg/mL, Sangon Biotech, China). The screened conjugated EC600 strain was verified by detection of the carbapenemase gene.

Whole genome sequencing (WGS) of CRKP strain was performed by Shanghai OE Biotech. PlasmidFinder 2.1 (https://cge.cbs.dtu.dk/services/Plasmid.Finder/) was used to obtain plasmid replication subtypes. IS Finder (https://www-is.biotoul.fr/blast.php) was used to query plasmid insertion sequences. ORF Finder (http://www.bioinformatics.org/sms/orf_find.html) was used to query open read boxes. Prokka was used for gene annotation of sequencing results, and CGView Server (http://cgview.ca/) was used to conduct online plasmid mapping.

### Isolation and characterization of OMVs from CRKP

OMVs were extracted by an electrophoresis and dialysis-based method (ELD) with a 300 kDa cut-off dialysis bag [17]. Specially, 200 ml of DMEM (Gibco, Shanghai, China) inoculated with the single colony was incubated at 37 ℃ with shaking at 200 rpm for 20–24 h. The culture solution was centrifuged at 10,000×*g* for 10 min to collect the culture supernatant, which was then filtered through a 0.22 μm filter (Beyotime, Shanghai, China) to remove bacteria and bacterial debris. Next, the OMVs of CRKP was isolated from filtered supernatant as previously described [17].

The OMVs of CRKP were photographed by transmission electron microscopy (TEM). Five µL of OMV suspension fixed with anhydrous ethanol was dropped onto a 200-mesh copper wire at room temperature for 1 min. Then, the sample was drained with filter paper and negatively dyed with 2% uranium acetate for 1 min. Afterwards, the excess dye was drained with filter paper. Then, the OMVs were observed by a Tecnai G2 Spirit Bio transmission electron microscope (FEI, America). NanoSight NS500 (Thermo Scientific, America) was used for determination of OMVs, and the particle size and concentration of OMVs were analyzed by NTA software.

### Proteomics analysis of CRKP and carbapenem-susceptible K. pneumoniae (CSKP) OMVs

After extracting OMVs from CRKP and CSKP strains by ELD, the two groups of the purified OMVs were sent to Shanghai Sangon Biotech for the detection of OMV proteins by liquid chromatography tandem-mass spectrometry (LC–MS/MS). All the nature and function of the proteins were determined at https://www.ncbi.nlm.nih.gov/guide/proteins/, which divided the proteins into 10 categories, including ABC transporter, ribosomal protein, enzyme, iron transport-related protein, lipoprotein, outer membrane protein, peptidoglycan related protein, DNA binding protein, uncharacteristic protein and others.

### Bioassay to determine carbapenem inactivation

Five groups were used for the bioassay to determine carbapenem inactivation by OMVs. The control group (group 1) contained only 0.9% NaCl and meropenem. Group 2 contained OMV suspensions and meropenem. Group 3 contained OMV suspensions, which was treated with 100 μg/mL proteinase K (Sangon Biotech, Shanghai, China) for 2 h at 37 °C to breakdown proteins, and proteinase K was deactivated with 10 mM phenylmethanesulfonyl fluoride (PMSF), followed by the addition of meropenem. Group 4 contained OMV suspensions, which was treated with 2% Triton X-100 to break OMVs, before adding meropenem. Group 5 contained OMV suspensions, which was treated with Triton X-100, proteinase K and PMSF before adding meropenem. Meropenem in these 5 groups was used at a concentration of 8 μg/mL. The 5 groups were incubated for 20 h at 37 °C. Afterwards, 20 μL of solution from each group was loaded onto a blank disk (Yankouzhongyi, China). These disks were placed on an MH agar plate coated with the ATCC 25922 strain, a carbapenem-susceptible *E*. *coli*. The plates were incubated overnight at 37 °C for 16–18 h, and the diameters of the inhibition zones were measured. A decreased diameter of inhibition zone compared to the control group (group 1) indicated carbapenem hydrolysis.

### Statistical analysis

The data were processed in Excel format and by GraphPad Prism 5 (GraphPad Software, CA, USA). It was considered statistically significant if the *p*-value was <0.05.

## Results

### The increasing isolation of carbapenem-resistant *Enterobacterales* in clinical settings

**Fig. 2:**
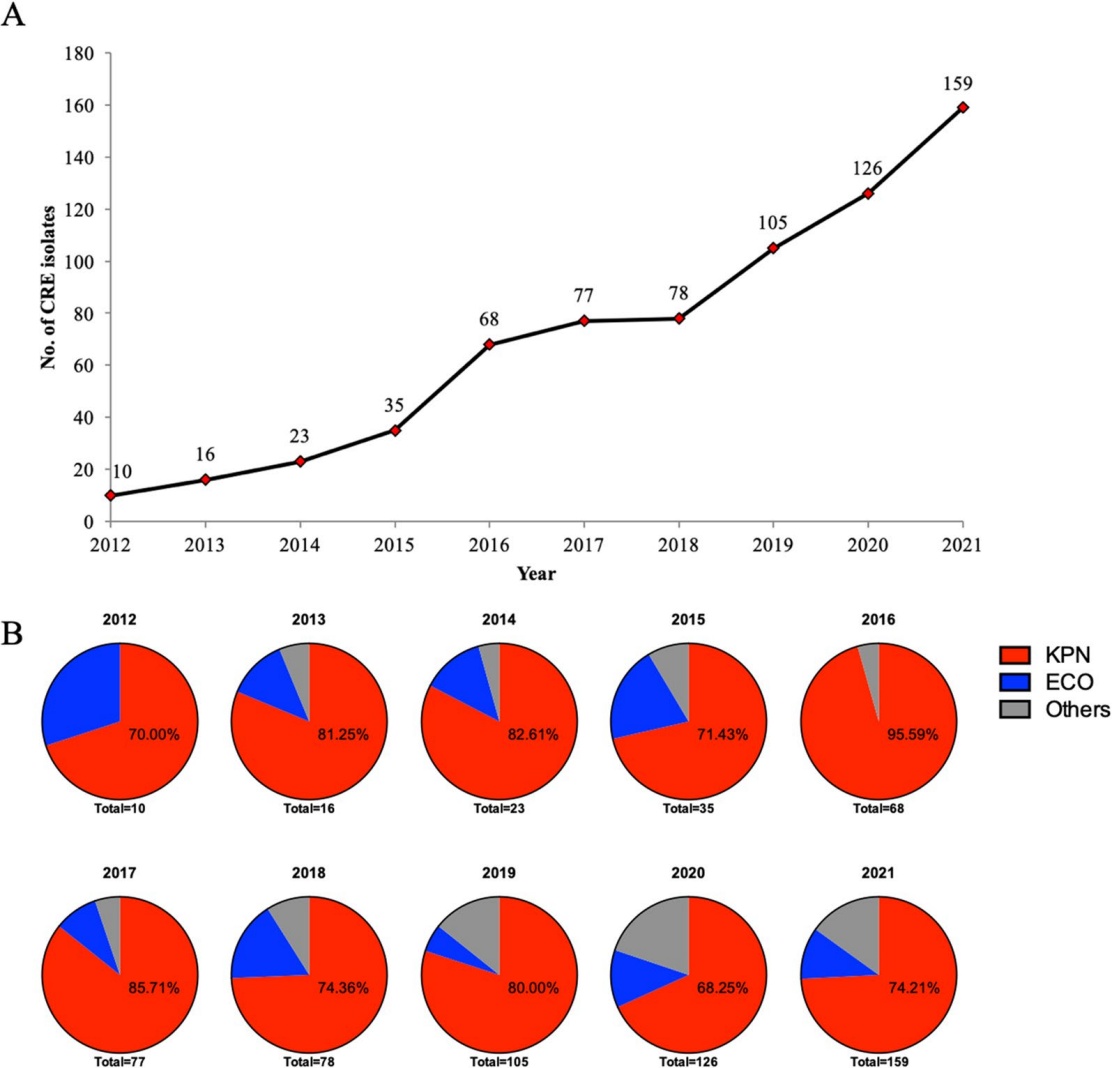
The status of carbapenem-resistant *Enterobacterales* in clinical settings. **a** The number of carbapenem-resistant *Enterobacterales* (CRE) isolates in a tertiary hospital from 2012 to 2021. **b** The composition of *Klebsiella pneumoniae* (KPN), *Escherichia coli* (ECO) and other species (others) among CRE isolates in a tertiary hospital from 2012 to 2021

By reviewing laboratory records, we found that the number of CRE isolates in this tertiary hospital from Shanghai increased from 10 to 159 over years from 2012 to 2021 (Fig. 2a). The sharp increase in the isolation of CRE in this hospital started in 2015 (Fig. 2a). By identifying the CRE isolates to the species level, we also found that carbapenem-resistant *K*. *pneumoniae* accounted for the majority of CRE in this tertiary hospital, with proportions as high as 95.59% of all CRE in 2016, followed by carbapenem-resistant *E*. *coli* (Fig. 2b). Notably, the percentage of species other than *K*. *pneumoniae* and *E*. *coli* has risen gradually in recent years (Fig. 2b). All these data indicated that the isolation of CRE isolates has increased in hospital settings.

### Characterization of carbapenemase genes in carbapenem-resistant *Enterobacterales*

**Fig. 3:**
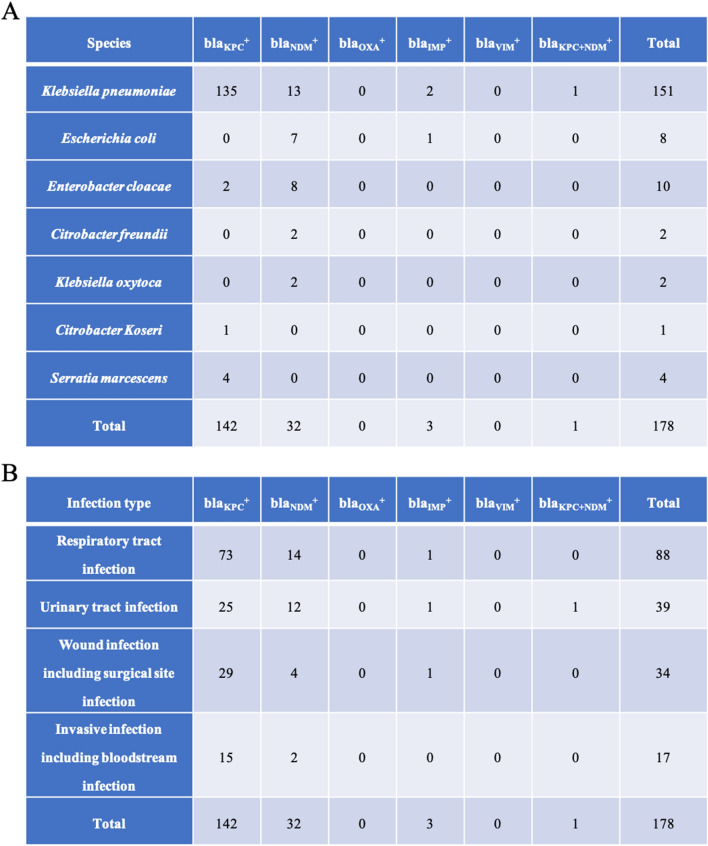
Distribution of carbapenemase genes in CRE isolates and infection types. **a** The distribution of carbapenemase genes in different CRE isolates. **b** The distribution of carbapenemase genes in different infection types

As a great increase in CRE isolates occurred in the year of 2015, the CRE isolates from 2015/07 to 2017/07 were enrolled for detection of carbapenemase genes. The CRE isolates from the other period from 2020/04 to 2021/04 were also utilized for representative of recent situation. After applying inclusion and exclusion criteria, a total of 178 CRE isolates causing clinical infections were collected, including 100 CRE isolates from 2015/07 to 2017/07 and 78 CRE isolates from 2020/04 to 2021/04. As shown in Fig. 3a, the bacterial species included 151 K. *pneumoniae* (84.8%), 8 *E*. *coli* (4.5%), 10 *Enterobacter cloacae* (5.6%), 2 *Citrobacter freundii* (1.1%), 2 *Klebsiella oxytoca* (1.1%), 1 *Citrobacter koseri* (0.6%) and 4 *Serratia marcescens* (2.2%). By the amplification of carbapenemase genes by PCR, including blaKPC, blaNDM, blaIMP, blaVIM and blaOXA, we found that the carbapenemase gene blaKPC encoding *Klebsiella pneumoniae* carbapenemase (KPC) was the most prevalent, followed by blaNDM. By combining the species distribution among CRE and the carbapenemase genes, it can be concluded that blaKPC-positive *K*. *pneumoniae* was the most prevalent strain in this hospital. The blaNDM-positive *K*. *pneumoniae* could also be isolated while the *E. coli* mainly carried blaNDM. The CRE isolates caused 88 cases of respiratory tract infection (RTI), 39 urinary tract infection (UTI), 34 wound infections including surgical site infection and 17 invasive infections including bloodstream infection. Then we explored the association between carbapenemase genes and infection type. As shown in Fig. 3b, the blaKPC-positive CRE isolates could cause all infections including RTI, UTI, wound infections and invasive infections, whereas the blaNDM-positive CRE isolates usually caused RTI and UTI. Interestingly, a *K*. *pneumoniae* strain co-producing KPC and NDM (JY03543) causing UTI was isolated. It was reported that the transmission of mobile elements carrying resistance genes was a major pathway by which MDROs increased, and then the 151 CRKP isolates were subjected to plasmid conjugation experiments. As a result, 37 CRKP isolates were successfully conjugated, including 32 strains of transconjugants expressing *bla*KPC and 5 strains of transconjugants expressing bla*NDM*. The transconjugants of the CRKP JY03543 strain, which contained blaKPC and blaNDM, expressed only blaNDM_,_ which suggested that the plasmid carrying blaNDM not the one carrying blaKPC in the CRKP JY03543 strain was transmissible.

### Prevalence of ST15 carbapenem-resistant *K*. *pneumoniae*

**Fig. 4:**
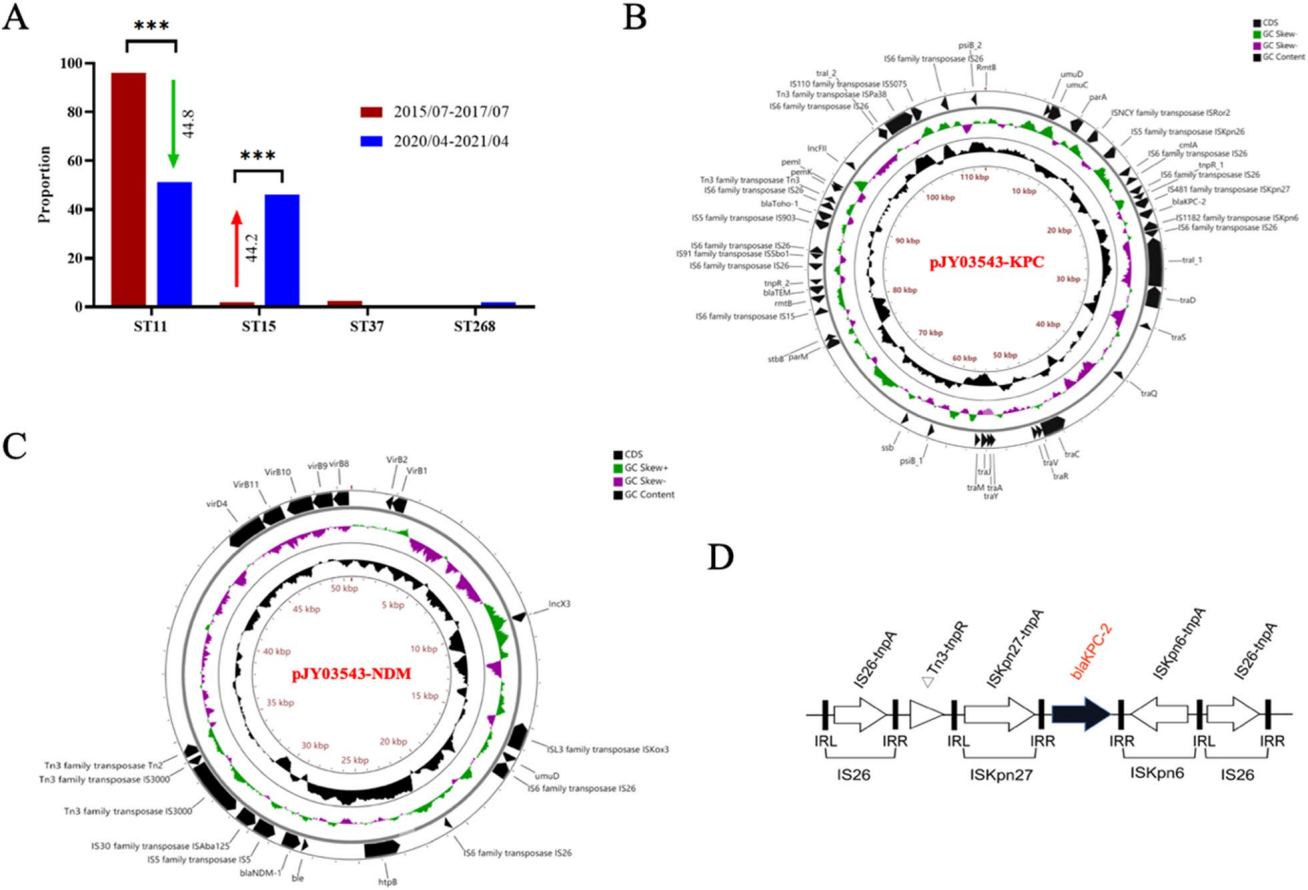
Molecular characterization of carbapenem-resistant *Klebsiella pneumoniae.* **a** The sequence type (ST) of CRKP in two distinct periods, expressed as means ± SD. ***p < 0.001. **b** The mapping of plasmid pJY03543-KPC carrying blaKPC-2. **c** The mapping of plasmid pJY03543-NDM carrying blaNDM-1. **d** The surrounding gene structure of blaKPC

The CRKP strains predominated among CRE isolates, and during the study we also found that the CRKP isolates were mainly isolated from the emergency department and surgical intensive care unit. Then we focused on the CRKP from these two units. Fifty-one CRKP and 39 CRKP isolates were collected from 2015/07 to 2017/07 and 2020/04 to 2021/04, respectively. By multilocus sequence typing, we found that ST11 CRKP (96.1%) was the leading type in the early period of 2015/07 to 2017/07 (Fig. 4a). With a dynamic change, the proportion of ST15 CRKP increased from 2.0% to 46.2% in the late period of 2020/04 to 2021/04, whereas the ST11 CRKP was still the major type (Fig. 4a). These data indicated that ST11 and ST15 CRKP co-existed as two prevalent types in these two units. The CRKP JY03543 strain also belonged to ST11. By whole genome sequencing of the strain JY03543, the results showed that the carbapenemase genes, blaKPC-2 and blaNDM-1, were located on two different plasmids, named pJY03543-KPC and pJY03543-NDM, respectively (Fig. 4b, c). As shown in Fig. 4b, the size of pJY03543-KPC was 112.1 kb, with a 53.0% average G + C content. The plasmid type of pJY03543-KPC was Inc FII, and a total of 394 open reading frames (ORFs) were predicted. As shown in Fig. 4c, pJY03543-NDM was 50.624 kb, with a 49.2% average G + C content, and the plasmid type was Inc X3 with a total of 176 ORFs. There were some protein genes associated with plasmid stability in pJY03543-KPC, such as *ParB*, *Umu CD*, *pem IK*, *Psi AB* and *ssb*. The plasmid conjugative transfer region mainly included the *Tra* and *Trb* conjugative transfer genes. In addition to blaKPC-2, blaToho-1, blaTEM and cmlA were also located on pJY03543-KPC (Fig. 4b). Tn3–ISKpn27–blaKPC–ΔISKpn6 was the core module of blaKPC-2, with IS26 inserted at both ends (Fig. 4d). The plasmid stability related region of pJY03543-NDM encoded the protein *Umu CD*, and the plasmid conjugated transfer region mainly included the VirB/D4 system gene. In addition to blaNDM-1, the bleomycin resistance gene (ble) was carried by pJY03543-NDM (Fig. 4c).

### Analysis of OMVs from CRKP

**Fig. 5:**
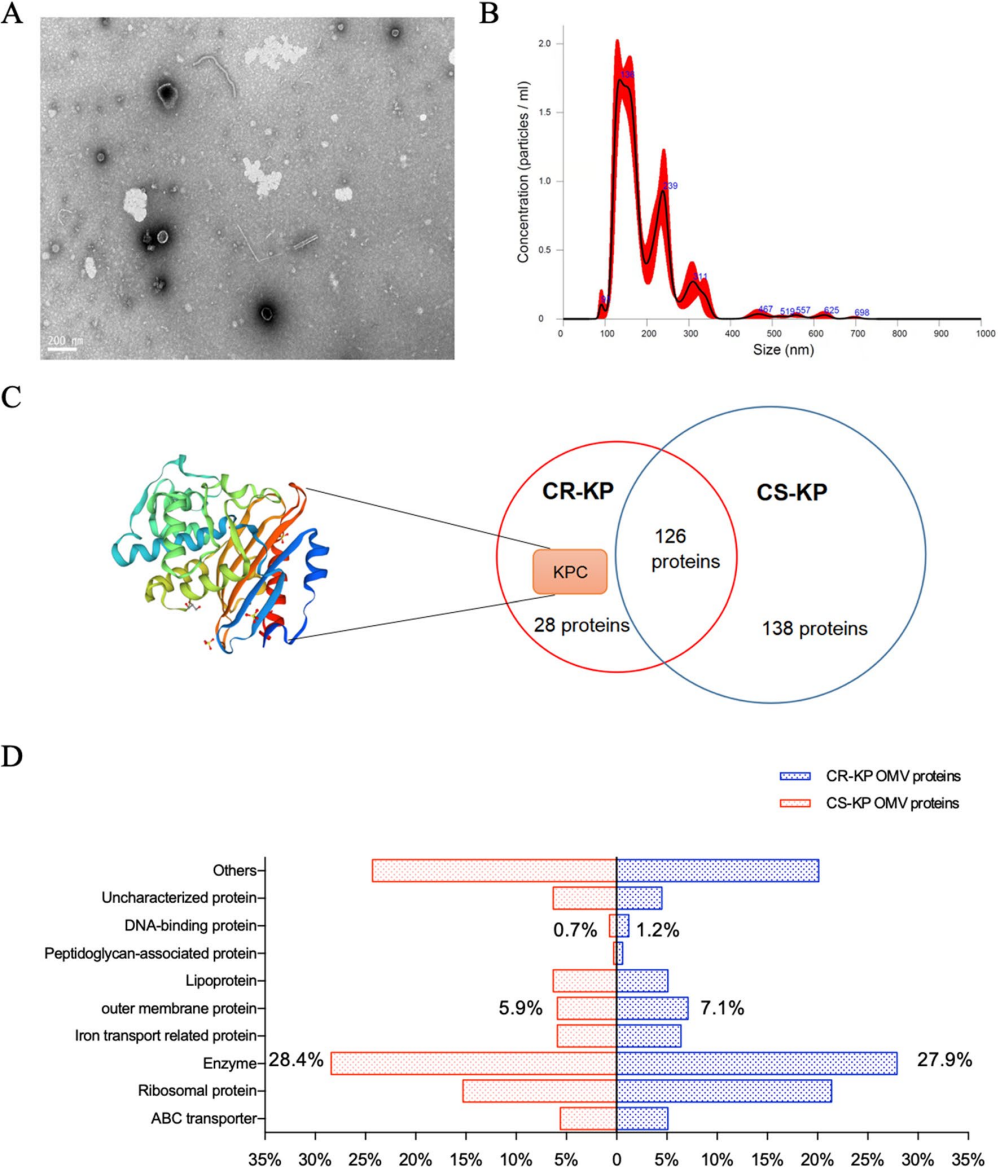
Isolation and analysis of OMVs from CRKP. **a** OMVs isolated from culture supernatant under transmission electron microscope (TEM). **b** Size distribution and concentration of OMVs measured with nano-particle tracking analysis. **c** Venn diagram of protein amounts of the CRKP and CSKP OMVs with identification of a characterful protein named KPC from CRKP. **d** Classification of the proteins from OMVs

The OMVs were isolated from the culture supernatant by an electrophoresis and dialysis-based method with a 300 kDa cut-off dialysis bag. Multiple sizes of bilayer membrane vesicle-like structures were observed under TEM (Fig. 5a). The particle size analysis of OMVs showed that the diameters of *K*. *pneumoniae* OMVs ranged from 100 to 200 nm (Fig. 5b). We selected OMVs from a CRKP strain and a CSKP strain to complete the analysis of *K*. *pneumoniae* OMV proteomics. Finally, 154 proteins were identified from the CRKP OMVs and 264 proteins were identified from the CSKP OMVs, with the discovery of a characteristic protein named KPC in the CRKP group, and the resulting Venn diagram was shown in Fig. 5c. The protein structure of KPC was also shown in Fig. 5c, with a size of 31.141 kD. To better understand the proteomics of OMVs from *K*. *pneumoniae*, we classified all the proteins into 10 types based on their properties and functions (Fig. 5d). Proteins from “enzyme” group, which participate in various physiological metabolic processes, were the most common type of protein within OMVs (accounting for 28.4% of CSKP OMV proteins, and 27.9% of CRKP OMV proteins).

### Meropenem can be hydrolyzed by OMV-associated KPC

**Fig. 6:**
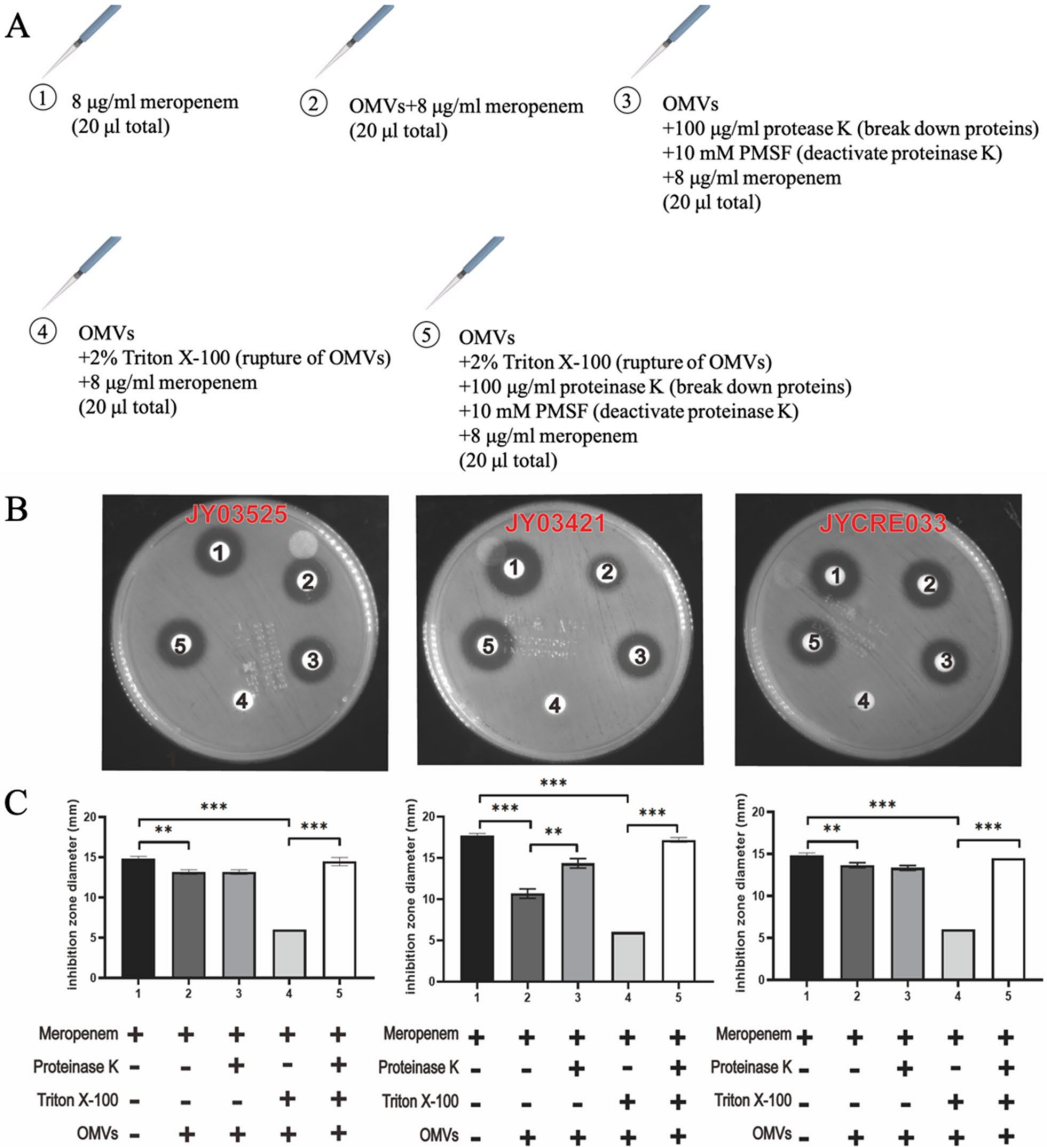
Meropenem can be hydrolyzed by OMV-associated KPC. **a** Disks from 1 to 5 contained indicated solutions. **b** Inhibition zones resulting from incubation of *E*. *coli* ATC25922, with 8 μg/mL meropenem exposure to free KPC or/and OMV-associated KPC, with or without pretreatment with proteinase K or/and Triton X-100. **c** Summarized data of diameters of inhibition zones in three independent experiments, expressed as means ± SD. **p < 0.01, ***p < 0.001

As KPC was detected in OMVs from CRKP, we tried to explore the role of OMVs in antimicrobial resistance. OMVs from three different blaKPC-positive CRKP strains were used for the meropenem hydrolyzing assay. Figure 6a showed five disks containing different solutions, and Fig. 6b showed the overall inhibition zones from three different OMVs with various treatments, and the summarized data were displayed in Fig. 6c. The results of group 2 in all three panels showed significant differences from those of group 1, indicating that there was free KPC in OMV suspensions from these three CRKP isolates. As proteinase K could degrade free enzymes, the results of group 3 in the left and right panels showed no changes compared to the results of group 2, implying a limited amount of free KPC in OMV suspensions from these two CRKP isolates. However, in the middle panel, the diameters of the inhibition zone in group 3 increased due to the degradation of free KPC by proteinase K treatment. These results suggested that the free KPC in the OMV solutions was susceptible to proteinase K degradation and the amount of free KPC in OMV suspensions varied from different CRKP isolates. In all three panels, the results of group 4 did not show any inhibition zone because meropenem was hydrolyzed by KPC enzyme released from OMVs by Triton X-100. The results of groups 1, 4 and 5 proved that the OMV-inside KPC can be protected from proteinase K degradation, and this protection was abolished in the presence of Triton X-100. These data suggested that meropenem can be hydrolyzed by OMV-associated KPC, which indicated a critical role of OMVs in antimicrobial resistance among CRKP.

## Discussion

The isolation rate of CRE in China has increased, and the same goes for Shanghai. Taking CRKP in Shanghai as an example, it reached the highest percentage level in 2019, with a slight downward trend from 2020 to 2021 (Fig. 1), which may be due to better methods of detection, surveillance, and antibiotic stewardship. The production of carbapenemases is the main resistance mechanism of CRE. KPC is the most prevalent carbapenemase in China, followed by NDM and IMP, while the carbapenemases VIM and OXA are relatively rare [3]. In 2006, the first case of *K*. *pneumoniae* producing KPC-2 in China was reported in Zhejiang Province [18]. In this study, we found that the main epidemic enzyme type in this tertiary hospital was KPC, followed by NDM, consistent with the domestic situation. In addition, we found blaIMP-positive strains in both periods, which has been prevalent in Japan [19, 20]. Therefore, the continued existence of blaIMP-positive strains should arise more attention. Regarding the strain co-producing both KPC and NDM, which was also confirmed by whole genome sequencing (WGS), it carried the blaKPC-encoding plasmid and the blaNDM-encoding plasmid. Many cases of this kind of CRE have been reported worldwide [21–23], which may be due to the mobile genetic elements, and some studies found that the more resistance to carbapenem antibiotics of double enzyme-producing strains has been developed compared to strains producing KPC or NDM [24]. Therefore, the emergence of blaKPC and blaNDM double positive CRE may bring new challenges to clinical treatment.

The molecular epidemiology of epidemic CRKP worldwide has shown regional differences. ST258 is the most common type in European and American countries [25], while ST11 is the dominant type in China [26]. According to the results of our study, in the period from 2015/07 to 2017/07, the prevalent CRKP strains in the surgical intensive care unit and emergency ward mostly belonged to ST11, accounting for 96.1%. During 2020/04–2021/04, the proportion of ST15 CRKP increased significantly from 2.0% to 46.2%. Meanwhile, the proportion of ST11 decreased from 96.1% to 51.3%, suggesting that ST15 CRKP has become a circulating type in this tertiary hospital. Similar results have been reported that ST15 CRKP became predominant in some hospitals in China [27–29]. Some researchers analyzed the difference in virulence between ST11 and ST15 and found that ST15 CRKP was associated with the KfuB virulence gene and more resistant to serum killing than the ST11 CRKP strain [28].

In addition to clone dissemination, mobile plasmids have played an important role in the widespread epidemic of CRE [4, 30, 31]. The ability of CRKP to transmit antimicrobial resistance through plasmids was explored by conjugation assay in our study, and 37 out of 151 isolates were successfully conjugated, indicating that the epidemic of CRKP in this hospital was partly due to plasmid transmission. Notably, only the blaNDM-positive plasmid was successfully conjugated after plasmid conjugation of the blaKPC and blaNDM double positive strain JY03543. After WGS analysis of this strain, it was found that blaKPC was located in the Inc FII plasmid and blaNDM was located in the smaller Inc X3 plasmid. It has been reported that Inc X3 is the most common type of Inc carrying the blaNDM gene [32, 33], and that the Inc X3 plasmid has a high conjugation frequency [34], which is more easily transferred to different bacteria. However, it is possible that the plasmid pJY03543-KPC has a conjugation function, because the plasmid structure still has the plasmid conjugation region (tra and trb). Further exploration of the mechanism underlying the emergence of multiple carbapenemases-producing strains would contribute to the control of CRE infections.

Our study identified the presence of KPC in OMVs from blaKPC-positive CRKP isolates, which can still hydrolyze meropenem in vitro. This phenomenon has also been demonstrated by a study from Zhang et al. [14]. In the field of microbiology, OMVs have attracted attention mainly for their role in bacterial virulence and as vaccines. The OMVs of *E*. *coli*, *Acinetobacter baumannii* and *Stenotrophomonas maltophiliahas* have been reported to play a role in antimicrobial resistance [35–37]. Our results confirmed that CRKP may be able to hydrolysis antibiotics by secreting OMVs loaded with meropenem-inactivating enzymes and found that OMVs have a protective effect on KPC from outside digestion, such as proteinase digestion. However, this protective effect disappeared when the membrane was broken by Triton X-100, suggesting that KPC was in the OMV cavity or anchored to the OMV membrane, similar to other OMV-related proteins [38, 39]. Proteomic studies of OMVs from CRKP and CSKP by LC–MS/MS also showed that OMVs carried DNA-binding proteins, which indicated the ability of OMVs to carry DNA. And researches have shown that OMVs generated from CRKP are able to induce the horizontal intraspecific transfer of drug resistance genes and virulence genes [40]. In summary, these results indicated that OMVs play a critical role in the antimicrobial resistance.

## Conclusions

In conclusion, the isolation rate of CRE is still on the rise, and appropriate interventions are in need for slowing down the spread of CRE. The molecular epidemiological characteristics of CRE from two different periods showed that KPC and NDM were the epidemic CRE-producing enzymes in this tertiary hospital. Meanwhile, the proportion of ST15 CRKP increased rapidly, which may indicate that the predominant CRKP type is dynamically changing. The results of the conjugation assay indicated that the prevalence of CRKP in this hospital was partly due to the transmission of plasmids. Analysis of CRKP OMVs suggested that OMVs may play a critical role in antimicrobial drug resistance, which provides important information for overcoming the CRE challenge.

## Data Availability

The data used and analyzed in this study is available from the corresponding author on reasonable request.

## Abbreviations

MDROs: Multi-drug resistant organisms
CRE: Carbapenem-resistant *Enterobacterales*
KPC: *Klebsiella pneumoniae* Carbapenemase
VIM: Verona vntegron-encoded metallo-beta-lactamase
IMP: Imipenemase
NDM: New Delhi metallo-beta-lactamase
OXA-48: Oxacillinase-48
RTI: Respiratory tract infection
UTI: Urinary tract infection
CRKP: Carbapenem-resistant *Klebsiella pneumoniae*
ST: Sequence type
EV: Extracellular vesicles
OMVs: Outer membrane vesicles
ESBL: Extended-spectrum beta-lactamase
CARSS: China Antimicrobial Resistance Surveillance System
MLST: Multilocus sequence typing
LB: Luria–Bertani
WGS: Whole genome sequencing
ELD: Electrophoresis and dialysis-based method
TEM: Transmission electron microscope
CSKP: Carbapenem-susceptible *Klebsiella pneumoniae*
LC–MS/MS: Liquid chromatography tandem-mass spectrometry
PMSF: Phenylmethanesulfonyl fluoride
CRKP/CR-KPN: Carbapenem-resistant *Klebsiella pneumoniae*
CR-ECO: Carbapenem-resistant *Escherichia coli*
ORFs: Open reading frames

## References

[1] Logan LK, Weinstein RA. The epidemiology of carbapenem-resistant enterobacteriaceae: the impact and evolution of a global menace. J Infect Dis. 2017;215(Suppl_1):S28–36. 10.1093/infdis/jiw282 PMC5853342 28375512

[2] van Duin D, Doi Y. The global epidemiology of carbapenemase-producing Enterobacteriaceae. Virulence. 2017;8(4):460–9. 10.1080/21505594.2016.1222343 PMC5477705 27593176

[3] Zhang R, Liu L, Zhou H, Chan EW, Li J, Fang Y (2017). Nationwide surveillance of clinical carbapenem-resistant enterobacteriaceae (CRE) strains in China. EBioMedicine.

[4] Zhang R, Chan EW, Zhou H, Chen S (2017). Prevalence and genetic characteristics of carbapenem-resistant Enterobacteriaceae strains in China. Lancet Infect Dis.

[5] Yang Q, Jia X, Zhou M, Zhang H, Yang W, Kudinha T (2020). Emergence of ST11-K47 and ST11-K64 hypervirulent carbapenem-resistant *Klebsiella pneumoniae* in bacterial liver abscesses from China: a molecular, biological, and epidemiological study. Emerg Microbes Infect.

[6] Zhou Y, Wu C, Wang B, Xu Y, Zhao H, Guo Y (2023). Characterization difference of typical KL1, KL2 and ST11-KL64 hypervirulent and carbapenem-resistant *Klebsiella pneumoniae*. Drug Resist Updates.

[7] Briaud P, Carroll RK (2020). Extracellular vesicle biogenesis and functions in gram-positive bacteria. Infect Immun..

[8] Deo P, Chow SH, Han ML, Speir M, Huang C, Schittenhelm RB (2020). Mitochondrial dysfunction caused by outer membrane vesicles from Gram-negative bacteria activates intrinsic apoptosis and inflammation. Nat Microbiol.

[9] Schwechheimer C, Kuehn MJ (2015). Outer-membrane vesicles from Gram-negative bacteria: biogenesis and functions. Nat Rev Microbiol.

[10] Kulkarni HM, Jagannadham MV (2014). Biogenesis and multifaceted roles of outer membrane vesicles from Gram-negative bacteria. Microbiology (Reading, England).

[11] Toyofuku M, Nomura N, Eberl L (2019). Types and origins of bacterial membrane vesicles. Nat Rev Microbiol.

[12] Ciofu O, Beveridge TJ, Kadurugamuwa J, Walther-Rasmussen J, Høiby N (2000). Chromosomal beta-lactamase is packaged into membrane vesicles and secreted from Pseudomonas aeruginosa. J Antimicrob Chemother.

[13] Martínez MMB, Bonomo RA, Vila AJ, Maffía PC, González LJ. On the offensive: the role of outer membrane vesicles in the successful dissemination of New Delhi metallo-β-lactamase (NDM-1). mBio. 2021;12(5):e0183621. 10.1128/mBio.01836-21 PMC8546644 34579567

[14] Zhang X, Qian C, Tang M, Zeng W, Kong J, Fu C (2023). Carbapenemase-loaded outer membrane vesicles protect Pseudomonas aeruginosa by degrading imipenem and promoting mutation of antimicrobial resistance gene. Drug Resist Updates.

[15] Poirel L, Walsh TR, Cuvillier V, Nordmann P (2011). Multiplex PCR for detection of acquired carbapenemase genes. Diagn Microbiol Infect Dis.

[16] Diancourt L, Passet V, Verhoef J, Grimont PA, Brisse S (2005). Multilocus sequence typing of *Klebsiella pneumoniae* nosocomial isolates. J Clin Microbiol.

[17] Yang M, Liu X, Luo Q, Xu L, Chen F (2020). An efficient method to isolate lemon derived extracellular vesicles for gastric cancer therapy. J Nanobiotechnology.

[18] Wei ZQ, Du XX, Yu YS, Shen P, Chen YG, Li LJ (2007). Plasmid-mediated KPC-2 in a *Klebsiella pneumoniae* isolate from China. Antimicrob Agents Chemother.

[19] Hayakawa K, Nakano R, Hase R, Shimatani M, Kato H, Hasumi J (2020). Comparison between IMP carbapenemase-producing Enterobacteriaceae and non-carbapenemase-producing Enterobacteriaceae: a multicentre prospective study of the clinical and molecular epidemiology of carbapenem-resistant Enterobacteriaceae. J Antimicrob Chemother.

[20] Hagiya H, Yamamoto N, Kawahara R, Akeda Y, Shanmugakani RK, Ueda A (2018). Risk factors for fecal carriage of IMP-6-producing Enterobacteriaceae at a long-term care hospital in Japan: a follow-up report from the northern Osaka multicentre study group. J Infect Chemother.

[21] Liu Y, Long D, Xiang TX, Du FL, Wei DD, Wan LG (2019). Whole genome assembly and functional portrait of hypervirulent extensively drug-resistant NDM-1 and KPC-2 co-producing *Klebsiella pneumoniae* of capsular serotype K2 and ST86. J Antimicrob Chemother.

[22] Liao W, De Wang L, Li D, Du FL, Long D, Liu Y (2021). High prevalence of 16s rRNA methylase genes among carbapenem-resistant hypervirulent *Klebsiella pneumoniae* isolates in a Chinese Tertiary Hospital. Microbial drug resistance (Larchmont, NY).

[23] Liu J, Du SX, Zhang JN, Liu SH, Zhou YY, Wang XR (2019). Spreading of extended-spectrum β-lactamase-producing Escherichia coli ST131 and *Klebsiella pneumoniae* ST11 in patients with pneumonia: a molecular epidemiological study. Chin Med J.

[24] Wu W, Espedido B, Feng Y, Zong Z (2016). Citrobacter freundii carrying blaKPC-2 and blaNDM-1: characterization by whole genome sequencing. Sci Rep.

[25] Marsh JW, Mustapha MM, Griffith MP, Evans DR, Ezeonwuka C, Pasculle AW (2019). Evolution of outbreak-causing carbapenem-resistant *Klebsiella pneumoniae* ST258 at a tertiary care hospital over 8 years. mBio.

[26] Liao W, Liu Y, Zhang W (2020). Virulence evolution, molecular mechanisms of resistance and prevalence of ST11 carbapenem-resistant *Klebsiella pneumoniae* in China: a review over the last 10 years. J Glob Antimicrob Resist.

[27] Han Y, Huang L, Liu C, Huang X, Zheng R, Lu Y (2021). Characterization of carbapenem-resistant *Klebsiella pneumoniae* ST15 clone coproducing KPC-2, CTX-M-15 and SHV-28 spread in an intensive care unit of a Tertiary Hospital. Infect Drug Resist.

[28] Chen J, Hu C, Wang R, Li F, Sun G, Yang M (2021). Shift in the dominant sequence type of carbapenem-resistant *Klebsiella pneumoniae* bloodstream infection from ST11 to ST15 at a Medical Center in Northeast China, 2015–2020. Infect Drug Resist.

[29] Shi Q, Han R, Guo Y, Zheng Y, Yang Y, Yin D (2020). Emergence of ST15 *Klebsiella pneumoniae* clinical isolates producing plasmids-mediated RmtF and OXA-232 in China. Infect Drug Resist.

[30] Schweizer C, Bischoff P, Bender J, Kola A, Gastmeier P, Hummel M (2019). Plasmid-mediated transmission of KPC-2 carbapenemase in enterobacteriaceae in critically ill patients. Front Microbiol.

[31] Tang Y, Zhou Y, Meng C, Huang Y, Jiang X (2020). Co-occurrence of a novel VIM-1 and FosA3-encoding multidrug-resistant plasmid and a KPC-2-encoding pKP048-like plasmid in a clinical isolate of *Klebsiella pneumoniae* sequence type 11. Infect Genet Evolut.

[32] Wu W, Feng Y, Tang G, Qiao F, McNally A, Zong Z (2019). NDM Metallo-β-lactamases and their bacterial producers in health care settings. Clin Microbiol Rev.

[33] An J, Guo L, Zhou L, Ma Y, Luo Y, Tao C (2016). NDM-producing Enterobacteriaceae in a Chinese hospital, 2014–2015: identification of NDM-producing Citrobacterwerkmanii and acquisition of blaNDM-1-carrying plasmid in vivo in a clinical Escherichia coli isolate. J Med Microbiol.

[34] Wang Y, Tong MK, Chow KH, Cheng VC, Tse CW, Wu AK (2018). Occurrence of highly conjugative IncX3 epidemic plasmid carrying bla (NDM) in Enterobacteriaceae isolates in geographically widespread areas. Front Microbiol.

[35] Bielaszewska M, Daniel O, Karch H, Mellmann A (2020). Dissemination of the blaCTX-M-15 gene among Enterobacteriaceae via outer membrane vesicles. J Antimicrob Chemother.

[36] Liao YT, Kuo SC, Chiang MH, Lee YT, Sung WC, Chen YH (2015). *Acinetobacter baumannii* Extracellular OXA-58 is primarily and selectively released via outer membrane vesicles after sec-dependent periplasmic translocation. Antimicrob Agents Chemother.

[37] Devos S, Stremersch S, Raemdonck K, Braeckmans K, Devreese B (2016). Intra- and interspecies effects of outer membrane vesicles from *Stenotrophomonas maltophilia* on β-lactam resistance. Antimicrob Agents Chemother.

[38] González LJ, Bahr G, Nakashige TG, Nolan EM, Bonomo RA, Vila AJ (2016). Membrane anchoring stabilizes and favors secretion of New Delhi metallo-β-lactamase. Nat Chem Biol.

[39] Wang X, Eagen WJ, Lee JC (2020). Orchestration of human macrophage NLRP3 inflammasome activation by Staphylococcus aureus extracellular vesicles. Proc Natl Acad Sci USA.

[40] Wang Z, Wen Z, Jiang M, Xia F, Wang M, Zhuge X (2022). Dissemination of virulence and resistance genes among *Klebsiella pneumoniae* via outer membrane vesicle: an important plasmid transfer mechanism to promote the emergence of carbapenem-resistant hypervirulent *Klebsiella pneumoniae*. Transbound Emerg Dis.

